# The Endocannabinoid System: A Putative Role in Neurodegenerative Diseases

**DOI:** 10.5812/ijhrba.9222

**Published:** 2013-12-14

**Authors:** Giuseppe Di Iorio, Matteo Lupi, Fabiola Sarchione, Ilaria Matarazzo, Rita Santacroce, Filippo Petruccelli, Giovanni Martinotti, Massimo Di Giannantonio

**Affiliations:** 1 Department of Neuroscience and Imaging, University G. d’Annunzio, Chieti, Italy; 2 Department of Human, Social and Health Sciences, University of Cassino, Cassino, Italy

**Keywords:** Endocannabinoids, Cannabinoid Receptors, Neurodegenerative Diseases

## Abstract

**Background:**

Following the characterization of the chemical structure of D9-tetrahydrocannabinol (THC), the main psychoactive constituent of marijuana, researchers have moved on with scientific valuable explorations.

**Objectives:**

The aim of this review is to highlight the role of endocannabinoid system in neurodegenerative diseases.

**Materials and Methods:**

The article is a critical analysis of the most recent data currently present in scientific literature on the subject; a qualitative synthesis of only the most significant articles has been performed.

**Results:**

In central nervous system, endocannabinoids show a neuromodulatory function, often of retrograde type. This way, they play an important role in synaptic plasticity and in cognitive, motor, sensory and affective processes. In addition, in some acute or chronic pathologies of central nervous system, such as neurodegenerative and neuroinflammatory diseases, endocannabinoids can perform a pro-homeostatic and neuroprotective function, through the activation of CB1 and CB2 receptors. Scientific evidence shows that an hypofunction or a dysregulation of the endocannabinoid system may be responsible for some of the symptoms of diseases such as multiple sclerosis, amyotrophic lateral sclerosis, Huntington’s, Parkinson’s and Alzheimer’s diseases.

**Conclusions:**

The important role played by endocannabinoid system promises interesting developments, in particular to evaluate the effectiveness of new drugs in both psychiatry and neurology.

## 1. Introduction

The use of marijuana for medical and recreational purposes can be identified in different historical periods and various cultures (1). Since early studies, which used extracts of *Cannabis sativa*, it has been possible to derive raw notions about the actions of this compound.

Following the characterization of the chemical structure of D9-tetrahydrocannabinol (THC), the main psychoactive constituent of marijuana (2), researchers have moved on with scientific valuable explorations. Understanding the mechanism of action of cannabinoids has consented extraordinary progresses, including the cloning and the expression of cannabinoid receptors (CB1 and CB2) in both rats and humans. Scientific research has thus been oriented towards the identification of endogenous ligands of these receptors: the so-called endocannabinoids. The endocannabinoid system consists not only of receptors and endogenous ligands but also of a complex apparatus for molecules synthesis and degradation (3, 4).

In general terms, the endocannabinoid system is involved in many physiological functions, many of which are related to neuroprotective and antinociceptive properties. Furthermore, the endocannabinoid system is involved in modulation of immune, inflammatory and endocrine responses (3-5).

## 2. Objectives

The aim of this review is to highlight the role of endocannabinoid system in neurodegenerative diseases

## 3. Materials and Methods

We searched Pubmed to identify published meta-analysis, reviews, randomized double-blind trials, open-label trials and case reports written in English, focusing on the role played by endocannabinoid system in neurodegeneration. The following keywords were used: endocannabinoid system, cannabinoid receptors, and neurodegenerative diseases. The search was conducted the 29th of July 2012 and yielded a total number of 138 results. After reading titles and abstracts, we excluded 36 articles from total record. Analyzing the full texts of the 102 remaining articles, we made a qualitative synthesis, reporting in this overview the most representative papers. Therefore, we searched Scopus, Google Scholar and PsycInfo in order to identify any other study missed by previous analysis. No further study has been evidenced using same keywords.

## 4.Results

In central nervous system, endocannabinoids show a neuromodulatory function, often of retrograde type. This way, they play an important role in synaptic plasticity and in cognitive, motor, sensory and affective processes. In addition, in some acute or chronic pathologies of central nervous system, such as neurodegenerative and neuroinflammatory diseases, endocannabinoids can perform a pro-homeostatic and neuroprotective function, through the activation of CB1 and CB2 receptors. Scientific evidence shows that an hypofunction or a dysregulation of the endocannabinoid system may be responsible for some of the symptoms of diseases such as multiple sclerosis, amyotrophic lateral sclerosis, Huntington’s, Parkinson’s and Alzheimer’s diseases. 

### 4.1. The Endocannabinoid System

Sixty different types of cannabinoids have been identified in Cannabis sativa; the two most represented are Δ9-tetrahydrocannabinol (THC) and cannabidiol (CBD). These molecules have different (and almost opposing) effects: while THC is psychotomimetic, CBD has antipsychotic and anxiolytic properties (6).

Modifications in these cannabinoids rates are then associated to variations in quality and intensity of the experience associated with cannabis consumption. THC, thanks to its high lipid solubility, can pass the blood-brain barrier; its psychoactive properties can activate endogenous cannabinoid receptors densely distributed throughout the brain.

The most important endogenous cannabinoids are anandamide (AEA) and 2-arachidonoyl-glycerol (2-AG). Unlike classical neurotransmitters, endocannabinoids are not stored in vesicles but are produced by neurons as soon as the body requires them, using lipidic constituents of the membrane. The hypothesis that endocannabinoids act as retrograde messengers is becoming more convincing; they could mediate intercellular signals from postsynaptic neurons back to pre-synaptic terminals, where they inhibit the release of neurotransmitters. At least two types of cannabinoid receptors have been identified: CB1 and CB2. After their release, endocannabinoids act on receptors and then are rapidly inactivated by re-uptake and enzymatic degradation system.

**Figure 1.**
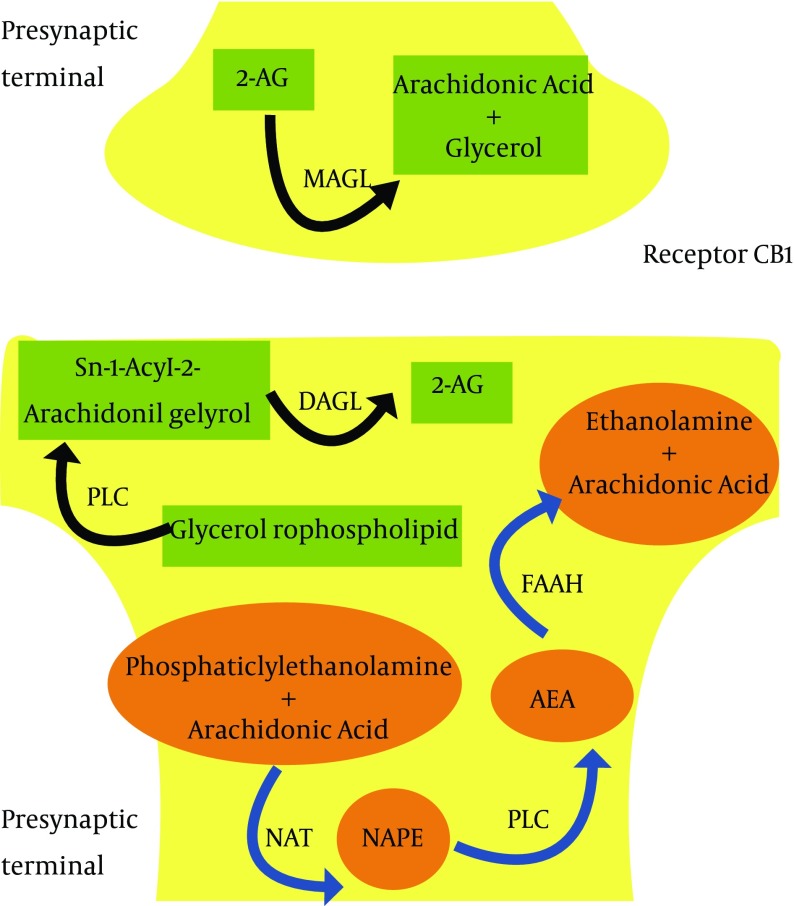
Anandamide (AEA) Synthesis Is Started by Enzyme N-acyltransferase (NAT), That Catalyzes the Reaction of Phosphatidyletanolamine With Arachidonic Acid to Produce N-Acylphosphatidyl-ethanolamine (NAPE), Which Is Converted by a Phospholipase D (PLD) in AEA; the Latter Is Inactivated by the Enzyme Fatty Acid Amide Hydrolase (FAAH). These Reactions Seem to Occur in Postsynaptic Terminal, While Catalyzer Enzymes for 2-arachidonoylglycerol (2-AG) Biosynthesis (Phospholipases C [PLC] and sn-1-selective Diacylglycerol Lipases [DAGL]) Are Peculiarly Localized in Postsynaptic Neurons. Monoacylglycerol Lipase (MAGL), Which Catalyzes 2-AG Inactivation, Is Instead Located in Presynaptic Neurons, Supporting a Possible Role as a Retrograde Messenger on Presynaptic CB1 Receptors for This Compound.

Enzymatic degradation system is performed by specific enzymes: FAAH (fatty acid amide hydrolase) and MAGL (monoacylglyceride lipase). FAAH is designed to degrade AEA, MAGL instead catalyzes 2-AG breakdown (7, 8) (Figure 1). 

These reactions seem to occur in postsynaptic terminal, while catalyzer enzymes for 2-AG biosynthesis (phospholipases C [PLC] and sn-1-selective diacylglycerol lipases [DAGL]) are peculiarly localized in postsynaptic neurons. Monoacylglycerol lipase (MAGL), which catalyzes 2-AG inactivation, is instead located in presynaptic neurons, supporting a possible role as a retrograde messenger on presynaptic CB1 receptors for this compound.

### 4.2. The Cannabinoid Receptors

The human body possesses specific binding sites for cannabinoids, distributed on the surface of many different cells. These receptors belong to the vast family of G protein-coupled receptors (GPCRs), which includes the majority of most common receptors. The GPCRs are membrane receptors consisting of seven trans-membrane domains (7TM) with an extracellular amino-terminal and an intracellular carbonyl terminal (9).

Cannabinoid receptors have different tissue distribution and mechanisms of signaling. CB1 are among the most abundant and widely distributed GPCRs in the brain. They can be found mainly on the nerve cells (neurons) in central nervous system (CNS). In the brain, the distribution of CB1 is particularly marked in the regions responsible for motor coordination and movement (for example, cerebellum, basal ganglia, striatum and substantia nigra), attention and complex cognitive functions, such as judgment (for example, cerebral cortex), learning, memory and emotions (for example, amygdala and hippocampus) (10, 11). In addition, CB1 receptors are present to a lesser extent in some organs and peripheral tissues, including endocrine glands, salivary glands, leukocytes, spleen, heart and part of the reproductive, urinary and gastrointestinal systems.

The distribution of CB1 receptors suggests a physiological role for endocannabinoids in the control of movements and perceptions, learning and memory processes, as well as in the regulation of emotional states such as pleasure and aggressiveness.

Unlike CB1, CB2 receptors are expressed primarily in immune cells and tissues, including leukocytes, spleen, tonsils, and bone marrow but also in the pancreas. They have recently been identified also in CNS, in particular on glial and microglial cells, albeit at low concentrations (12). 

The role of cannabinoid receptors is essentially to regulate the release of other chemical messengers. CB1 receptors interfere with the release of certain transmitters: their activation protects the CNS from overstimulation or over-inhibition that may be caused by other neurotransmitters.

CB2 receptors play instead a predominantly peripheral role with immunomodulatory activities. One of the functions of cannabinoid receptors is, in fact, to modulate the release of cytokines, protein molecules responsible for the regulation of immune function and inflammatory responses. Cannabinoids, therefore, may have an impact on neurodegenerative diseases through two main ways, neuro- and immunomodulation.

### 4.3. Endocannabinoids in Neurodegenerative Diseases

Scientific evidence shows that cannabis can provide symptomatic relief in several neurodegenerative diseases such as multiple sclerosis, Huntington’s, Parkinson’s and Alzheimer’s diseases, and amyotrophic lateral sclerosis. These findings imply that a hypofunction or a dysregulation of the endocannabinoid system may be responsible for some of the symptoms of these diseases. Moreover, given the abundance of CB1 receptors in areas associated with movement and executive thought, researchers’ interest has often focused on endocannabinoid levels in patients with motor degenerative disorders.

The two main endocannabinoids involved in these mechanisms are, as already said, the AEA and 2-AG. Their pharmacological properties were initially considered similar, as the molecules were believed to be mutually exchangeable and almost indistinguishable in the regulation of synaptic functions, synaptic plasticity and in behavioral aspects, such as learning, memory, reward, addiction, antinociception, and anxiety. Evidence now suggests that AEA and 2-AG possess specific pharmacological properties, are engaged in different forms of synaptic plasticity and take part in different behavioral functions (13).

#### 4.3.1. Multiple Sclerosis

Multiple sclerosis (MS) is an important neurological disease that can affect both central and peripheral nervous system. It is characterized by inflammatory scattered injuries, essentially demyelinating, that do not spare axons.

Its etiology is not so clear, but we can assume that genetic and environmental factors cooperate in the development of this disease thanks to some evidences, for example that viral infections act on a genetically susceptible population activating the immune response towards myelin self-peptides (14-16). Obviously, the immune system activation involves the release of reactive oxygen and nitrogen species, such as proteases and cytotoxic/cytostatic cytokines, by immune cells, which mediate the inflammatory damage (17, 18); a possible therapeutic approach for MS treatment may be, though, immune response modulation.

Since 1980s, it has been seen that cannabinoid agonists could be effective in CNS demyelinating pathologies as anti-inflammatory drug (19). Thank to experimental demyelination models it is clear the evidence of cannabinoids therapeutic benefits.

Scientific researchers have demonstrated that activation of both CB1 and CB2 receptors reduces the intensity of deficits such as spasticity, tremor or neuropathic pain; CB2 receptors activation, in addition, regulates the disease progression connected with the inflammatory process (20).

MS therapy could therefore be founded on strategies aiming to reduce or slow down the demyelination and neurodegeneration processes, peculiar of this disease. The synthetic cannabinoid agonists HU210 or WIN 55212-2 protect oligodendrocytes from apoptosis induced by trophic elements deprivation, acting on both CB1 and CB2 receptors; they suppress the production of inflammatory molecules, like IL-1b, TNF-a and NO, by astrocytes and microglial cells (21, 22), as well as they enhance the release of anti-inflammatory cytokines IL-4, IL-10, IL-6 and interleukin-1 receptor antagonist (IL-1ra) (23, 24); finally, cannabinoid receptors activation has protective effects on neurons and oligodendrocytes and, attenuating pro-inflammatory mediators, suppresses chronic inflammatory responses.

#### 4.3.2. Huntington’s Disease

Huntington’s disease (HD) is an autosomal dominant inherited neurodegenerative disorder characterized by involuntary choreiform movements, cognitive impairment, metabolic abnormalities, and a relentlessly progressive course culminating in death 10–25 years after onset (25).

The genetic basis of HD is the expansion of a CAG trinucleotide repeated within the Huntingtin (HTT) gene, resulting in the production of HTT protein containing an expanded glutamine tract. This altered peptide is resistant to normal cellular processes of protein turnover and ‘‘aggregates’’ or ‘‘inclusions’’ of the aberrant protein accumulate within neurons in HD brain regions.

In Huntington's chorea pallidus-striatal fibers are damaged firstly; they contribute to produce CB1 receptors, whose levels decrease from the first onset of symptoms and are not sufficient to play a protective role (26).

Characteristic changes in CB1 and CB2 receptors in HD have been investigated. In the globus pallidus, initially, GABA/enkephalin efferent terminals degeneration determinates CB1 loss in the external segment (27), while in the internal segment an important CB1 loss is primarily found in co-localized receptors or in GABA/substance P neuronal pathology (28). On the other hand, different postmortem HD studies demonstrated that CB2 receptors are up-regulated in the striatum (29).

Analyzing lymphocyte preparations of HD patients, it has been seen that AEA levels were six-fold higher than those of control patients; this can be explained through the inhibition of FAAH function in AEA metabolism (30).

So the question to be answered is how much CB1 activation might be therapeutic in HD patients. Rodent lesion models show conflicting results about whether agonism on CB1 is neuroprotective, exacerbatory, or useful in the treatment of HD symptoms because it is not clear if the loss of these receptors could preclude their therapeutic use (31). In contrast, CB2 receptors present in the striatum increase prior to symptom-onset (29). Moreover, selective CB2 agonist has been shown to reduce neuronal loss through suppression of glial activation (29, 32). One interesting therapeutic option yet to be explored in HD may be growth factor stimulation of endogenous neurogenesis.

#### 4.3.3. Parkinson’s Disease

Parkinson’s disease (PD) is a degenerative illness of the CNS caused by death of dopaminergic neurons in the substantia nigra; this determines an insufficient formation and action of dopamine, producing a decreased motor cortex stimulation by basal ganglia. The discriminating traits of the disease are divided into primary symptoms, such as muscle rigidity, tremors and slowing of physical movements (bradykinesia), and secondary symptoms, such as a high level of cognitive dysfunction and subtle language problems. 

The neuromodulatory effects of endocannabinoid system are correlated with dopaminergic system, which in turn exerts a reciprocal regulation upon the endocannabinoids; it is not a coincidence that CB1 and D1/D2-like receptors are co-localized in striatal neurons (33, 34) and exhibit complex signalling interactions (35-37). For example, AEA has been shown to reduce dopamine release in striatal slice cultures and instead increasing it in nucleus accumbens in vivo (38, 39); moreover, D2 receptors activation has been noticed to increase AEA levels in the basal ganglia (40, 41).

An important contrast worthy to be evidenced is that, in parkinsonian tissue, the CB1 mRNA level has been shown to be decreased in caudate nucleus, anterior dorsal putamen and external segment of the globus pallidus, while an increase in CB1 binding in caudate nucleus and putamen has been observed by others studies (42, 43). These results and the effects demonstrated in all patients who underwent drug treatment are hard to interpret. Only one study investigated the endocannabinoids levels in PD patients, showing that AEA level in their cerebrospinal fluid was more than twice as of controls (44).

Different studies on the potential therapeutic usefulness of cannabinoid agonists and antagonists in PD have produced conflicting results; for example, it is not clear if cannabinoid antagonists could alleviate or not the typical Parkinson motor deficits (33, 45, 46), because some researches have demonstrated their failure, while others have shown that motor activity was improved with rimonabant (a CB1 inverse agonist) (47).

However, CB1 activation may alleviate a disabling motor complication resulting from long-term use of levodopa, the levodopa-induced dyskinesia (LID) (43) probably due to CB1-mediated alterations in dopamine and glutamate release (48).

#### 4.3.4. Alzheimer’s Disease

Alzheimer’s disease (AD) is the most common form of dementia, a disabling neurodegenerative disease that begins predominantly in subjects over 65 years of age (49).

From an etiological point of view, it is due to both genetic and idiopathic causes that imply a gross atrophy of neurons projecting to cerebral cortex and hippocampus, and also of their glutamatergic neurons (50). As a consequence, it is determined an extracellular deposition of b-amyloid protein in “plaques” and/or the formation of intracellular “tangles” of hyperphosphorylated Tau protein, causing the neurodegeneration.

The endocannabinoid system has recently raised a great deal of interest as a powerful modulator of neuronal activity (i.e. glutamatergic neurons) or inflammatory processes (51, 52). 

Studies on human AD brain have found CB1 expression on neurons reduced (53) or unchanged (54); in contrast, CB2 expression is dramatically up-regulated, particularly in the microglial cells surrounding b-amyloid plaques (53, 54).

Despite the challenge of targeting receptors that may potentially disrupt learning and memory, neuroprotective approaches have been taken to circumvent those effects by targeting more specifically CB2 receptor by modulating the degradation pathway of endocannabinoids, or by using low, non-psychoactive doses of non-selective agonists of CB1/CB2 receptors (55-57). It has been demonstrated indeed that endocannabinoids may mediate neuroprotection through activation of CB1 and may improve inhibition the inflammatory microglial response through activation of CB2 (53); CB2 agonists have been shown to inhibit TNF-a and nitric oxide production by microglia/macrophages, as well as stimulating their phagocytosis of b-amyloid peptide (58, 59). Hence, modulation of the endocannabinoid system in recently diagnosed AD patients by daily management of low doses of cannabinoids could at minimum delay the progression of the disease, i.e. reducing inflammation, sustaining potential for neurogenesis, reducing hyperphosphorylation of Tau and delaying memory impairment.

#### 4.3.5. Amyotrophic Lateral Sclerosis

In ALS, degeneration of motor neurons in cortex, brainstem and spinal cord is the most important element (60). The etiopathological mechanisms focus on neuroinflammation, mostly mediated by excitotoxicity and oxidative damage on motor neurons (61-63). Experimentally, in human ALS patients' spinal cord demonstrates motor neurons damages marked by CB2-positive microglia/macrophages (64). Treatment of ALS mouse models with D9-THC has showed an improvement of the symptoms for administration of the molecule either before or after signs onset (65).

Studies have demonstrated that CB1 deletion in ALS mice, while not altering motor neuron survival, extended lifespan by 15 days, a 13% increase in survival (66). For the future strategies, it’s important defining CB2 role and CB1-CB2 relationship. CB2 activation blocks b-amyloid induced microglia activation (53); but, on the other hand, with other stimuli, CB2 activation is showed increasing microglial migration and proliferation (67, 68).

Different studies in ALS mice have been conducted demonstrating that the CB2 agonist use can slow disease progression if administered after disease onset (69). Another study showed an increase of 56% of the survival interval (70). Moreover, analyzing the activated microglia from spinal cord in human ALS patients it has been seen a CB2 increase (64). So all these data show how modifying CB2-mediated processes could change ALS progression and how much the endocannabinoid system is potentially involved reducing neuroinflammation, so excitotoxic and oxidative cell damage (64, 71).

## 5. Conclusions

We can affirm that brain areas exchange information through a network of signals generated by neurotransmitters. Endogenous cannabinoids or endocannabinoids are part of this signaling mechanism: their action is similar to that of extracts from Indian hemp (cannabis) as hashish and marijuana. The endocannabinoid system is activated in several inflammatory and degenerative diseases of the brain, presumably to curb neuronal damage. Endocannabinoid system is often recruited in order to mitigate neuronal damage and inflammation, initially in areas peculiarly affected by the pathology, such as spinal cord in multiple sclerosis, basal ganglia (striatum and globus pallidus) in Parkinson's disease, and hippocampus and cerebral cortex in Alzheimer's disease. In Huntington's chorea, instead, pallidus-striatal fibers are damaged in the first instance: they are related to generation of CB1, whose levels decrease from the first onset of the symptoms and appear to be not sufficient to play a protective role. An overarching paradigm in the diseases summarized in this review is that hypofunction or dysregulation of the endocannabinoid system may be responsible for some of the symptomatology of these diseases.

Further studies are needed to completely understand endocannabinoid implications in neurodegenerative disorders, especially in regard to their possible implications in the therapy of above mentioned diseases. It has to be taken into account that the activation of cannabinoid receptors, in particular CB1, in brain areas and tissues other than those affected by the disease, can cause significant side effects, such as psychotropic effects typical of certain cannabis preparations.

Moreover, the possibility of cannabinoids to determine the onset of psychiatric disorders, such as psychotic episodes (72-75), panic attacks, mood swings, anhedonia (74), amotivational syndrome, impulsivity (76), self-harm (77) and multiple substance abuse (78, 79) must be taken into account and fully considered before starting a therapeutic strategy.

Finally it will be important use selective strategies for therapeutic exploitation of endocannabinoids with particular attention for example to synthetic molecules that selectively activate the CB2 receptors without a psychotropic by-play.
